# DMD Pluripotent Stem Cell Derived Cardiac Cells Recapitulate *in vitro* Human Cardiac Pathophysiology

**DOI:** 10.3389/fbioe.2020.00535

**Published:** 2020-06-19

**Authors:** Sarka Jelinkova, Aleksandra Vilotic, Jan Pribyl, Franck Aimond, Anton Salykin, Ivana Acimovic, Martin Pesl, Guido Caluori, Simon Klimovic, Tomas Urban, Hana Dobrovolna, Vladimir Soska, Petr Skladal, Alain Lacampagne, Petr Dvorak, Albano C. Meli, Vladimir Rotrekl

**Affiliations:** ^1^Department of Biology, Faculty of Medicine, Masaryk University, Brno, Czechia; ^2^International Clinical Research Center ICRC, St. Anne's University Hospital Brno, Brno, Czechia; ^3^CEITEC, Masaryk University, Brno, Czechia; ^4^PhyMedExp, University of Montpellier, INSERM, CNRS, Montpellier, France; ^5^First Department of Internal Medicine—Cardioangiology, Faculty of Medicine, Masaryk University, Brno, Czechia; ^6^Department of Biochemistry, Faculty of Science, Masaryk University, Brno, Czechia; ^7^Department of Clinical Biochemistry, St. Anne's University Hospital of Brno, Brno, Czechia; ^8^Second Clinic of Internal Medicine, Masaryk University of Brno, Brno, Czechia

**Keywords:** duchenne muscular dystrophy, DMD, human pluripotent stem cells, cardiomyocytes, intracellular calcium, excitation-contraction coupling, adrenergic response, cardiomyocyte death

## Abstract

Duchenne muscular dystrophy (DMD) is a severe genetic disorder characterized by the lack of functional dystrophin. DMD is associated with progressive dilated cardiomyopathy, eventually leading to heart failure as the main cause of death in DMD patients. Although several molecular mechanisms leading to the DMD cardiomyocyte (DMD-CM) death were described, mostly in mouse model, no suitable human CM model was until recently available together with proper clarification of the DMD-CM phenotype and delay in cardiac symptoms manifestation. We obtained several independent dystrophin-deficient human pluripotent stem cell (hPSC) lines from DMD patients and CRISPR/Cas9-generated DMD gene mutation. We differentiated DMD-hPSC into cardiac cells (CC) creating a human DMD-CC disease model. We observed that mutation-carrying cells were less prone to differentiate into CCs. DMD-CCs demonstrated an enhanced cell death rate in time. Furthermore, ion channel expression was altered in terms of potassium (Kir2.1 overexpression) and calcium handling (dihydropyridine receptor overexpression). DMD-CCs exhibited increased time of calcium transient rising compared to aged-matched control, suggesting mishandling of calcium release. We observed mechanical impairment (hypocontractility), bradycardia, increased heart rate variability, and blunted β-adrenergic response connected with remodeling of β-adrenergic receptors expression in DMD-CCs. Overall, these results indicated that our DMD-CC models are functionally affected by dystrophin-deficiency associated and recapitulate functional defects and cardiac wasting observed in the disease. It offers an accurate tool to study human cardiomyopathy progression and test therapies *in vitro*.

**Graphical Abstract:**
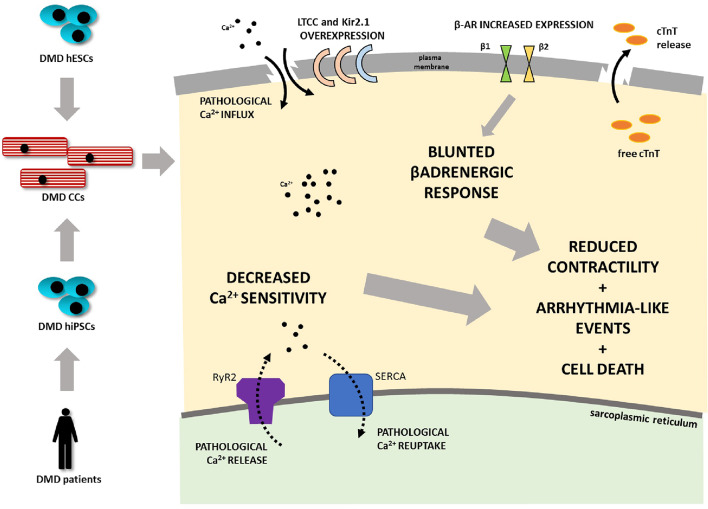
Duchenne muscular dystrophy (DMD) is associated with progressive dilated cardiomyopathy eventually leading to heart failure as the main cause of death in DMD patients. A human cardiomyocyte (CM) model was developed from several independent dystrophin-deficient human pluripotent stem cell (hPSC) lines from DMD patients and hESC line with deletion of DMD gene generated by CRISPR/Cas9 technology. DMD hPSC were differentiated into CMs. DMD mutation-carrying cells are less prone to differentiate into CMs. DMD CMs further demonstrate an enhanced cell death rate. Ion channel expression was altered in terms of potassium (Kir2.1 overexpression) and calcium handling (DHPR overexpression). DMD-CMs exhibited mishandling of calcium demonstrated by increased time of calcium release. Further mechanical impairment (hypocontractility), bradycardia, increased beat rate variability, and blunted β-adrenergic response connected with remodeling of β-adrenergic receptors' expression was found in DMD-CMs (LTCC L-type calcium channel, cTnT - cardiac troponin T, Kir2.1 - potassium channel).

## Introduction

Duchenne muscular dystrophy (DMD, prevalence 1/3,500–6,000 boys) is an X-linked genetic disorder which impairs striated muscles and results in severe disability and premature death in young men (Mah et al., 2014; Vry et al., 2016; Stehlíková et al., 2017). DMD is often associated with dilated cardiomyopathy (Manzur et al., 2008), which develops in affected patients during their teenage years. It is represented by a slowly progressing decline in diastolic function, systolic ejection fraction, and fractional shortening (Markham et al., 2006). Mechanical degeneration is associated with progressive cardiomyocyte (CM) wasting and spreading of fibrosis throughout the ventricular wall (Finsterer and Stöllberger, 2003; Panovský et al., 2019), which leads to intracardiac conduction disturbances, inducing atrial and life-threatening ventricular arrhythmias (Chenard et al., 1993; Himmrich et al., 2000). Gradual dilatation of the ventricle, thinning of the wall (Wagner et al., 2007), and consequent loss of contractility lead to repeated episodes of heart failure, which recently became the most frequent mortality cause in DMD patients worldwide (Finsterer and Stöllberger, 2003; Fayssoil et al., 2010).

DMD is caused by mutations in the dystrophin gene (*DMD*), which can result in a total lack of functional dystrophin or presence of a truncated inactive form of the protein (Blake et al., 2002). Dystrophin plays a major role in the organization of dystrophin glycoprotein complex (Clarac et al., 2009) responsible for sarcolemmal adhesion to the basal lamina and intracellular pathway regulations. Whereas, skeletal muscle degeneration is extensively studied, the cellular mechanisms of the cardiac pathophysiology are still unclear. Available studies point out the role of oxidative stress and DMD-induced Ca^2+^ mismanagement (Williams and Allen, 2007; Jung et al., 2008; Caluori et al., 2019), with subsequent cytosolic imbalance and cell damage (Gerke et al., 2005).

In DMD (muscular dystrophy on X chromosome, *mdx)* mice, we have shown that dystrophin deficiency caused post-translational remodeling of the cardiac ryanodine receptor (RyR2) macromolecular complex, due to S-nitrosylation and Calstabin2 (FKBP12.6) depletion, which leads to intracellular diastolic Ca^2+^ leak and ventricular arrhythmias (Fauconnier et al., 2010).

Intracellular Ca^2+^ leak from the sarcoplasmic reticulum (SR) was also associated with deregulation of stretch-activated channels (SAC) though reactive oxygen species (ROS)-mediated hyperactivity (Jung et al., 2008).

Further studies in animal models showed misregulation of other ion channels such as voltage-gated Nav1.5 and L-type calcium channel (LTCC) hyperactivation (Koenig et al., 2011), as well as upregulation of inflammation-related induced nitric oxide synthase (iNOS) (Fauconnier et al., 2010; Peterson et al., 2018). Such observations remain to be validated in human DMD. Furthermore, to this day, there are only limited studies of human DMD cardiac cell (CC) models, describing some of the discrepancies eventually leading to DMD-CM damage (Eisen et al., 2019; Pioner et al., 2020). In the present study, we thus focused, for the first time, on evaluating the impact of dystrophin-deficiency on some molecular properties of the excitation-contraction coupling (ECC) and flight-or-fight response in some patient-specific DMD human pluripotent stem cell-derived cardiac cells (DMD-hPSC-CCs) as well as in CRISPR/Cas9 engineered hPSC-CCs (summarized in Graphical abstract).

## Materials and Methods

**Figure 1:**
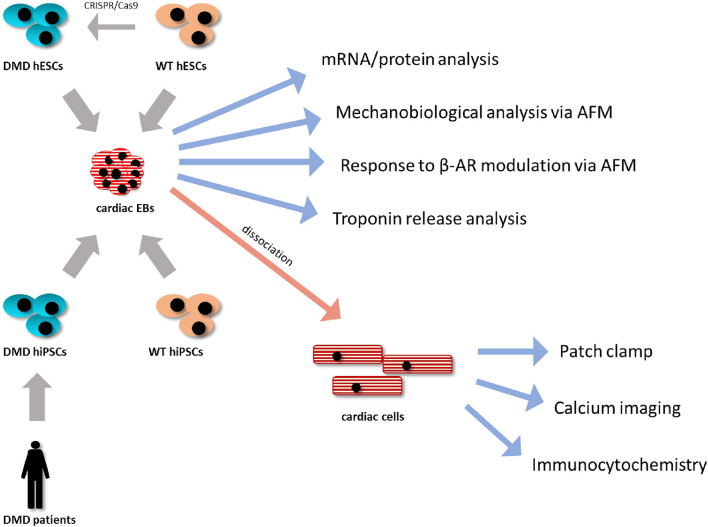
Methodological approach. DMD-hiPSC lines from two Duchenne muscular dystrophy (DMD) patients were generated and analyzed compared to WT-hiPSC lines. DMD-hESC line was generated using CRISPR/Cas9 technology by targetted deletion of the DMD gene from a healthy WT hESC line serving as isogenic control. All hPSC lines were differentiated using 3D cell aggregates (embryonic bodies, EBs). These EBs were then used for molecular and mechanobiological analysis methods or were enzymatically dissociated into isolated cardiac cells, which were further analyzed using single cell specific analysis as protein localization and ion fluxes analyses.

A wide selection of methods was used for analysis of the model starting from generation of the hPSC lines to analysis of 3D contracting clusters and dissociated CCs; thus, the methodological approach has been summarized in Figure 1.

### Cell Lines, Cultivation, Reprogramming, and hPSC Differentiation Into Cardiomyocytes via Embryoid Bodies

Patient-specific DMD human induced pluripotent stem cell (hiPSC) lines (DMD02 and DMD03) and CRISPR/Cas9 dystrophin-deficient human embryonic stem cell (hESC) line (cDMD) were used (described in Jelinkova et al., 2019a,b). The fibroblasts of two DMD patients were derived from skin/muscle (for DMD02/DMD03, respectively) biopsies. Informed consents approved by Ethics Committee (Faculty of Medicine, Masaryk University) were signed by parents of the patients beforehand and the investigation conformed to the principles outlined in the Declaration of Helsinki.

Control human embryonic stem cell (hESC) line CCTL14 (serving as isogenic control for cDMD) as well as CCTL12 hESC line (further referred to as WT hESC) derived in Masaryk University, Brno, and previously characterized (International Stem Cell Initiative et al., 2007), were used. hiPSC control line UEFhfiPS1.4 (Qu et al., 2013) and cl.4 obtained from Majlinda Lako (NewCastle, UK) (further called WT-hiPSC) were used for comparison with DMD-hiPSC to reduce the effect of reprogramming on the CC phenotype.

All human pluripotent stem cell (hPSC) lines were routinely maintained on feeder layer of mitotically inactivated mouse embryonic fibroblasts (mEF) as previously described (Dvorak et al., 2005; Krutá et al., 2014; Jelinkova et al., 2019a).

Cardiac differentiation and eventual subsequent dissociation of hPSC-CCs were performed for confocal fluorescent intracellular Ca^2+^ measurements, patch-clamp, atomic force microscopy (AFM), and immunocytochemistry as previously described (Pesl et al., 2014) with minor modifications. Briefly, grown colonies were cut into pieces with a pipette tip and clumps were transferred into differentiating embryonic body (EB) medium (86% KnockOut Dulbecco modified eagle medium, 10% fetal bovine serum, 1% L-glutamine, 1% penicillin/streptomycin, 1% nonessential amino acids, 0,1mM 2-mercaptoethanol) with 10 ng/ml bone morphogenic protein 4 (BMP4, R&D) and 10 μg/ml ascorbic acid (Sigma Aldrich) where they formed EBs. After 2 days, the medium was changed into EB medium with 5 ng/ml basic fibroblast growth factor (FGF2, Peprotech), 10 ng/ml BMP4, 6 ng/ml Activin A (R&D) and 10 μg/ml ascorbic acid (Sigma Aldrich) and incubated for 4 days. The next 3 days consisted of EB medium supplemented with 10 ng/ml vascular endothelial growth factor (VEGF, R&D), 10 μM IWR1 (Sigma Aldrich), and 10 μg/ml ascorbic acid. The EBs were further cultivated in EB medium with 10 μg/ml ascorbic acid, 10 ng/ml VEGF, and 5 ng/ml FGF2 for 12 days with medium changed every 4 days, and then with EB medium with 10 μg/ml ascorbic acid until further analysis. The differentiation procedure was performed for 13 days in hypoxic conditions (5% oxygen). Then, the EBs were moved into normoxic conditions (20% oxygen) for further cultivation.

### Immunodetection

Immunocytological analyses utilized [antibody specific against N-terminal (1:50 ICC/1:50-1:300 WB, Cat# NCL-DYSB, Leica Biosystems), and antibody specific against C-terminal (1:100 ICC/1:1000 WB, Abcam, Cat# ab15277)], for cardiac markers cardiac troponin T (cTnT; SantaCruz, Cat# sc-8121, 1:200) α-actinin (SigmaAldrich, Cat# A7180, 1:500) L-type channel (LTCC, dihydropyridine receptor DHPR alpha 2 subunit/CACNA2D1 antibody abcam, Cat# ab2864, 1:200), assays were performed as described previously (Krutá et al., 2013). For cTnT and α-actinin, cells were fixed using 4% paraformaldehyde at room temperature for 10-15 min. Cells for dystrophin labeling were fixed with 2% paraformaldehyde for 10 min at room temperature, then with ice cold methanol for 5 min, and incubated with pre-extraction buffer (25 mM HEPES, 50 mM NaCl, 1 mM EDTA, 3 mM MgCl2, 200 mM sucrose, 0.5% Triton X-100) before continuing to blocking steps.

The cells were permeabilized and blocked with blocking solution 1 (1% bovine serum albumin, BSA, 0.2% Triton X-100 in phosphate buffer saline, PBS, 15 min on ice) and blocking solution 2 (1% BSA, 1% NaN3, 0.05% Tween 20 in PBS, 60 min on ice). Primary antibodies were diluted in PBS/Tween (0.05%) and incubated overnight at 4°C. Samples were extensively washed with PBS/Tween (0.05%) and incubated with appropriate fluorescent secondary antibody for 1 h at room temperature. For nuclear counterstain, 4′,6-Diamidine-2′-phenylindole dihydrochloride (DAPI) was used. All fluorescent and confocal microscopy experiments were performed using Confocal LSM700 microscope and processed using Zen software (both Carl Zeiss, Oberkochen, Germany).

For dystrophin immunoblots, protein samples from cell lysates were run in 4–12% gradient polyacrylamide gel for 70 min/170V/400mA, blotted at 100 V/300 mA for 2 h, and labeled with antibody against dystrophin protein. After dystrophin detection, the membrane was incubated with antibody against lamin B as loading control (1:1000, Santa Cruz, sc6217). Adrenergic receptors (AR) were analyzed using β1 specific (Thermo Fisher, PA5-28808, 1:500) and β2 specific (abcam, ab137494, 1:500) antibody. Human heart atrial tissue was obtained as procedural surplus material, available during bicaval orthotopic heart transplantation from donor organs and was used as positive control as previously described (Pesl et al., 2020).

### Quantitative Real-Time PCR

Cardiac EBs 30–50 days old and human heart samples (obtained as surplus material from donor hearts during cardiac transplantations) were lysed using RNA Blue reagent (Top-Bio, Prague, Czech Republic) according to the manufacturer's instructions, and the total mRNA was isolated using the RNeasy Micro Kit (Qiagen, Hilden, Germany). RNA concentration and purity were determined using NanoDrop (NanoDrop technologies, Wilmington, DE, USA). cDNA was synthesized by Moloney Mouse Leukemia Virus (M-MLV) reverse transcriptase (Invitrogen, Carlsbad, CA, USA) at 37°C for 1 h followed by 5 min at 85°C. For quantitative real-time PCR (qRT-PCR), LightCycler® 480 SYBR Green Master kit (Roche, Basel, Switzerland) was used according to manufacturer's instructions, and PCR was performed, with annealing temperature 60°C and 55 cycles on LightCycler LC480 Instrument (Roche). The PCR primers (Generi-Biotech, Hradec Kralove, Czech Republic) are shown in Supplementary Table 1.

### Measurements of Intracellular Cytosolic Ca^2+^ Variations by Fluorescent Confocal Microscopy

The contracting EBs were dissociated to single CCs by incubation in Ca^2+^-free solution [120 mM NaCl, 5.4 mM KCl, 5 mM MgSO_4_, 5 mM sodium pyruvate, 20 mM glucose, 20 mM taurine, 10 mM HEPES (all Sigma Aldrich), pH6.9] at room temperature and enzymatically treated with 0.8 mg/ml type II collagenase (Gibco) and 0.037 mg/ml type XIV proteinase (Sigma Aldrich) in the same solution for 3 min at 37°C. The clusters were then incubated in dissociating solution (85 mM KCl, 30 mM K_2_HPO_4_, 5 mM MgSO_4_, 1 mM EGTA, 5 mM sodium pyruvate, 5 mM creatine, 20 mM taurine, 20 mM glucose, 2 mM disodium pyruvate, pH = 7.4) for 20 min at 37°C and plated on gelatin coated 35 mm glass bottom culture dishes (MatTek) in EB medium until the next day. On the day of measurements, cells were incubated in a Tyrode's solution (140 mM NaCl, 5 mM HEPES, 4 mM KCl, 1 mM MgCl_2_, 1.8 mM CaCl_2_, 10 mM glucose, pH 7.4) with 3 μM Fluo4-AM (Invitrogen, Carlsbad, CA, USA) for 20 min at room temperature. After the incubation with Fluo4-AM, the solution was replaced with the fresh Tyrode's solution and the cells were placed on the stage of an inverted microscope. Ca^2+^ images in line-scan mode were recorded with a laser scanning confocal microscope Zeiss LSM Exciter (Carl Zeiss), with 40x water immersion objective, in x–y mode, 1 image/0.495 s. Electrical pacing was set up with frequency 0.5, 1 Hz (20 V, 0.5 ms duration and 1 ms delay). The kinetic properties of intracellular Ca^2+^ variations including Ca^2+^ transients and diastolic events were analyzed by in-house developed PeakInspector software (https://asalykin.github.io/PeakInspector/) as previously described (Acimovic et al., 2018).

### Electrophysiological Recordings Through Patch-Clamp

Electrophysiological recordings were obtained using whole-cell patch-clamp technique on an Axopatch 200B (Axon Instruments, Foster City, CA) at room temperature (22–24°C). The recording pipettes contained: 120 mM KCl, 8 mM EGTA, 10 mM HEPES, 6.8 mM MgCl_2_, 3 mM CaCl_2_, 4 mM ATPNa_2_, 0.4 mM GTPNa_2_ (pH 7.2). The bath solution contained: 130 mmol/L NaCl, 4 mM/L KCl, 1.8 mM/L MgCl_2_, 1.8 mM/L CaCl_2_, 10 mM/L HEPES, 11 mM/L glucose (pH 7.4). Action potentials (APs) were measured in the current-clamp configuration in response to brief (1–2 ms) depolarizing current injections at 1 Hz. The resting membrane potential, the amplitude and duration (APDs) at 20, 50, and 90% repolarization of the APs were measured (respectively indicated as APD_20_, APD_50_, and APD_90_). 7 DMD-hPSC-derived CCs (DMD-CCs) and 18 WT-hPSC-derived CCs (WT-CCs) were analyzed. Cells arrhythmogenicity was tested using application of successive 10 s runs in the current clamp configuration defined by 5 brief (1 ms) depolarizing current injections at 2 Hz followed by a resting period. Data were analyzed using the Clampfit 10.0 (Axon Instruments) software.

### Atomic Force Microscopy Measurements

Atomic force microscope (JPK NanoWizard 3, JPK, Berlin, Germany), combined with an inverted light microscope (IX-81S1F-3, Olympus, Tokyo, Japan), was used to obtain mechanocardiograms of beating EBs as previously described (Pesl et al., 2014; Caluori et al., 2019; Pribyl et al., 2019). Drug response tests were performed after initial equilibration in Tyrode's solution, followed by addition of 70 μM metoprolol, 10 min of stabilization time, and then 10 min of measurement. Isoproterenol (1 μM) was subsequently added into the solution, and mechanocardiograms were measured after 20 min of stabilization. The mechanical properties of beating EBs were expressed as force of contraction in nN as previously described (Pesl et al., 2014); however, the values were relativized for final comparison.

### Free Troponin Analysis

The EBs were formed and cardiac induction was initiated as described above. On day 18, individual EBs homogenous in size and shape were separated onto 12 well plate coated with gelatin and 1ml of proper induction medium was added. From day 21 to 51 with frequency of 4 days, medium sample was collected, spun, and the supernatant frozen in −20°C until all samples were harvested. The samples were then analyzed for free troponin T concentration using two epitopes based immunochemical system Cobas e602 (Basel, Switzerland) specifically high sensitive short turnaround time (STAT) kit Elecsys Tropo T hs STAT 100 (Roche diagnostics, Basel, Switzerland; Hess et al., 2008) according to the manufacturer's instructions.

### Statistical Analysis

Statistical evaluation was carried out in GraphPad Prism 8.3 software (GraphPad Software, Inc., La Jolla, CA, USA). Available normality tests were performed for the obtained data, and Student's two-tailed *t*-test or ANOVA was used to assess statistically significant differences on normally distributed group pairs. Where normality tests failed, non-parametric tests were used. Individual statistical test used was specified in individual figure legends.

## Results

### DMD-CCs Do Not Express Full-Length Dystrophin Protein

**Figure 2:**
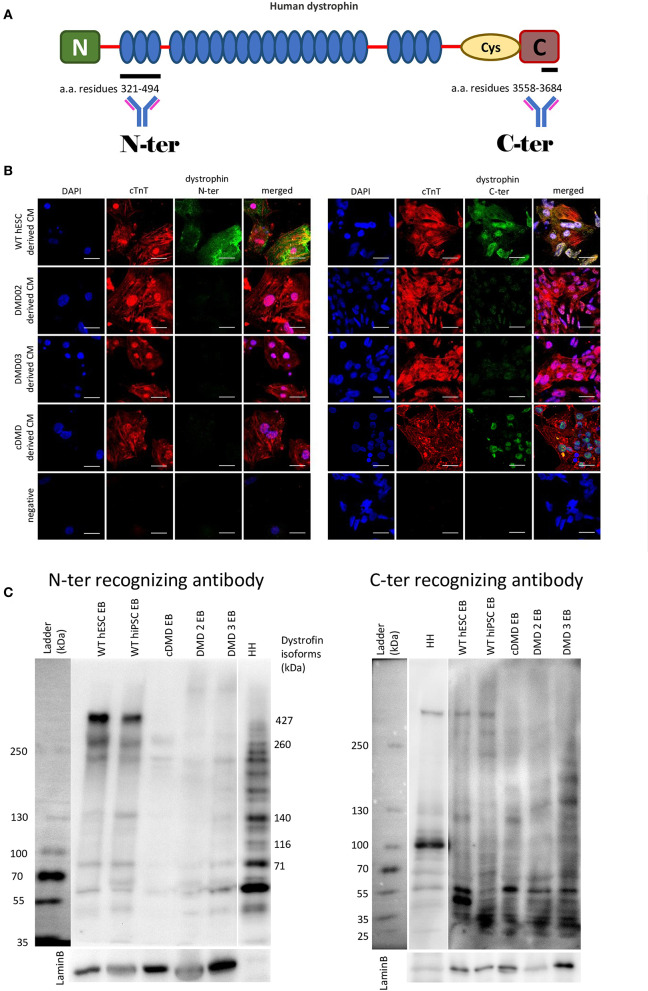
DMD-hPSC differentiate into cardiac cells (CCs), which do not express dystrophin. **(A)** Antibodies against dystrophin N-terminal (N) aminoacid (a.a.) residues 321–494 and C-terminal aminoacid residues 3558–3684 were used. **(B)** hPSC derived CCs were analyzed for presence of dystrophin (in green). WT hESC derived CCs dissociated from spontaneously beating EBs presenting with positive signal for cardiac troponin T (red) showed positive signal for dystrophin labeled by N-terminus specific antibody as well as by C-terminus specific antibody. None of the DMD-hPSC lines (DMD02, DMD03, and cDMD) derived CCs showed any signal for dystrophin. DAPI was used for nuclear counterstain. Ruler represents 50 μm. **(C)** The differentiated EBs were lyzed and western blot analysis was performed to identify the individual isoforms expressed. Both used WT-hPSC derived EBs show expression of various dystrophin isoforms including Dp427, Dp260, Dp140, and Dp71. The CRISPR/Cas9 generated cDMD-EBs show no expression of either isoform, while only shorter isoforms were identified in patient DMD-hiPSC-EBs with promoters downstream of the patient's mutations. Human heart tissue (HH) was used as positive control. LaminB was used as loading control.

Three previously described patient-specific DMD-hiPSC and one CRISPR/Cas9-mediated DMD-hESC lines (Jelinkova et al., 2019a,b) were differentiated into CMs (Pesl et al., 2014). WT hESC [hESC CCTL12 (hPSCreg name MUNIe005-A, passages 15–61) and CCTL14 (hPSCreg name MUNIe007-A, passages 15–63) derived in Masaryk University, Brno, and characterized previously (International Stem Cell Initiative et al., 2007)] and WT-hiPSC [cl.4 (passages 43–84) obtained from Dr. Majlinda Lako (Newcastle University, UK) (Armstrong et al., 2010) and hfiPSC (passages 20–52) (Qu et al., 2013)] were used as controls. The obtained DMD-CCs were evaluated for dystrophin presence. The strategy for antibody detection is illustrated in Figure 2A. Dystrophin expression was evaluated using immunocytology with specific antibodies against high molecular weight isoform of dystrophin (antibody specific to N-terminus detecting the full-length 427 kDa isoform, Figure 2B, left panel), as well as shorter isoforms (antibody specific to C-terminus, which is present in various lower molecular weight dystrophin isoforms, Figure 2B, right panel). Dystrophin expression was observed with both antibodies in WT-CCs as membranous signal of the antibody (Supplementary Figure 2). No membranous/cytoplasmic protein signal was detected in any DMD-CCs (Figure 2B). Nuclear signal of dystrophin was detected by C-ter antibody (which can detect shorter dystrophin isoforms, most likely the Dp71 isoform), and no cytoplasmic/membranous signal was observed in CCs derived from either DMD-hPSC lines. Furthermore, western blot analysis with N-ter and C-ter antibodies showed expression of some shorter dystrophin isoforms (Dp140, Dp116, and Dp71) in the DMD cell lines, but none in the full high molecular band (Dp427) (Figure 2C, full membranes in Supplementary Figure 2).

### Dystrophin Deficiency Leads to Cardiac Differentiation With Lower Yield of Spontaneously Beating EBs

**Figure 3:**
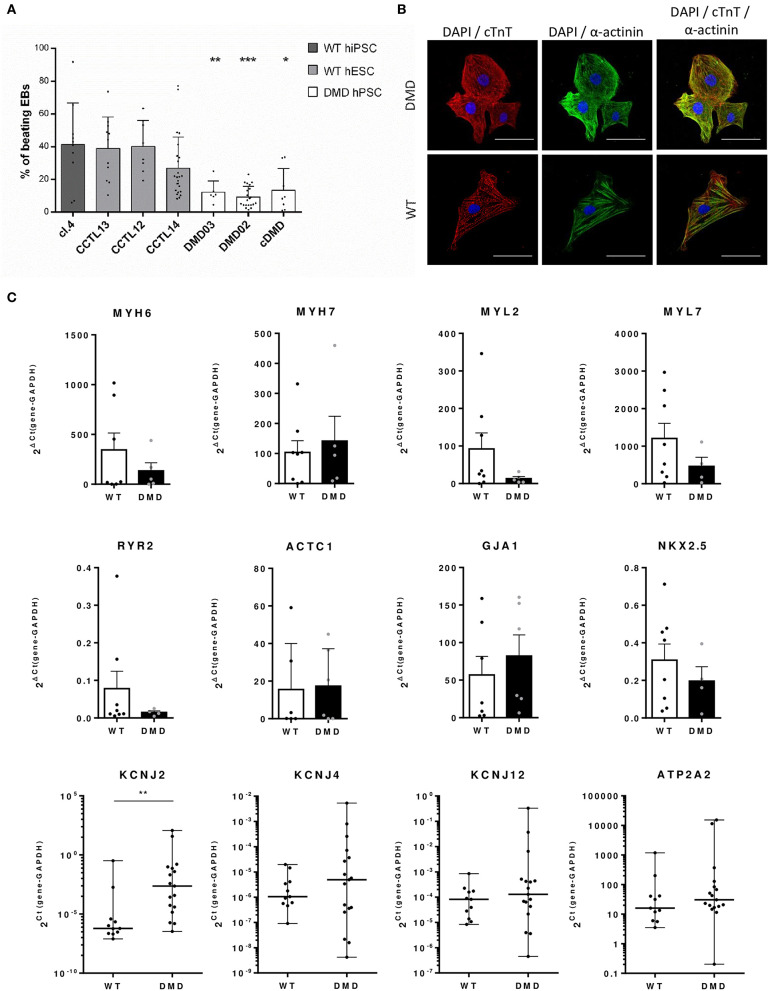
Differentiation efficacy is lower in DMD-hPSC lines. **(A)** All DMD-hPSC and WT-hPSC lines were differentiated into spontaneously contracting EBs containing cardiomyocytes and cardiac cells. The ratio of these spontaneously contracting clusters was evaluated and compared to wild-type (WT) controls (*n* = 36 for CCTL14 EBs, *n* = 8 for CCTL12 EBs, *n* = 13 for CCTL13, *n* = 12 for cl.4 EBs, *n* = 25 for DMD02 EBs, *n* = 6 for DMD03 EBs, *n* = 19 for cDMD-EBs). Lesser yields of beating EBs were identified in all DMD-hPSC lines. Statistical difference was calculated by one-way ANOVA and multiple comparison with Dunn's test. Significance (*p < 0.05, **p < 0.01, ***p < 0.01) is visualized as asterisks for the lowest values; for individual *p*-values, see Supplementary Table 1. **(B)** EBs were dissociated and cells labeled for cardiac markers α-actinin (in green) and cardiac troponin T (cTnT, in red) (DAPI is labeling the nuclei). WT and DMD-CCs presented similar level of typical striated pattern of sarcomeres. Line represents 50 μm. **(C)** Differentiation efficacy was evaluated by rt qPCR on mRNA levels of cardiac markers showing only non-significant differences in expression of various markers (MYH6, *n* = 7 for WT and *n* = 5 for DMD; MYL2, *n* = 8 for WT and *n* = 4 for DMD; MYL7, *n* = 8 for WT and *n* = 4 for DMD; RYR2, *n* = 8 for WT and *n* = 4 for DMD; ACTC1 *n* = 6 for WT and *n* = 5 for DMD; NKX2.5, *n* = 8 for WT and *n* = 4 for DMD, ATP2A2, *n* = 11 for WT and *n* = 17 for DMD) and non-significant increase in MYH7 (*n* = 8 for WT and *n* = 5 for DMD) and GJA1 (*n* = 7 for WT and *n* = 6 for DMD). Potassium channels were analyzed using Kir2.x isoforms mRNA expression (KCNJ2 – Kir2.1, KCNJ4 – Kir2.2 and KCNJ12 – Kir2.3, for all *n* = 11 for WT and *n* = 17 for DMD). Statistical significance on pooled DMD and pooled WT data sets was calculated using Mann–Whitney test; exact value for each is represented by • in each graph.

Next, we aimed at characterizing the impact of dystrophin deficiency on the cardiac differentiation of hPSCs through the EB-based method as previously published (Pesl et al., 2014). We compared the yield of beating EBs upon differentiation of all DMD and WT-hPSC lines. First of all, we used the spontaneous beat in 3D EB clusters as a criterion to evaluate the cardiac differentiation success. The differentiation efficacy represented by the fraction of spontaneously beating EBs (detected at day 29 after cardiac differentiation start) was decreased in DMD-EBs compared to WT-EBs (Figure 3A, statistical evaluation in Supplementary Table 2). At the cellular level, the beating EBs from all tested cell lines contained CCs (Figure 3B) with striated pattern of cardiac troponin T (cTnT) and α-actinin. The differentiation efficacy was also measured in age-matched groups of DMD- and WT-EBs aged 25–29 days by qRT-PCR of well-known cardiac markers [*NKX2.5 (p* = 0.42*)*, myosin heavy chain α (*MYH6, p* = 0.71), myosin heavy chain β (*MYH7, p* = 0.82), myosin light chain 2 (*MYL2, p* = 0.17), myosin light chain 7 (*MYL7, p* = 0.42), ryanodine receptor 2 (*RYR2, p* = 0.61), cardiac muscle alpha actin (*ACTC1, p* = 0.78), connexin43 (*GJA1, p* = 0.36)], showing no significant difference in relative mRNA expression. However, a tendency for lower yield of most of the markers in the obtained DMD clusters was observed, with the exception for *MYH7* and *GJA1* (Figure 3C). Inward rectifier potassium channels Kir2.1, Kir2.2, and Kir2.3 were also tested (*KCNJ2, KCNJ4*, and *KCNJ12*, respectively) with significant increase in Kir2.1 expression (*p* = 0.004); SERCA (*ATP2A*) shows no difference in mRNA expression.

### DMD-CCs Display Altered Electrophysiological Properties

**Figure 4:**
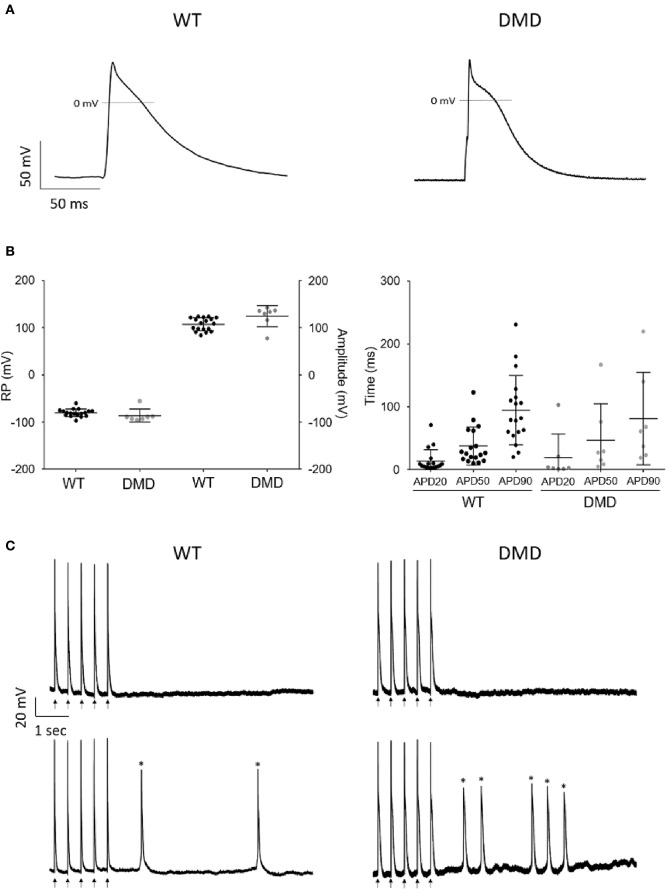
AP recordings showed no difference in WT and DMD-CCs. **(A)** Representative AP recordings obtained from WT and DMD-hiPSC derived CCs, under current clamp configuration. **(B)** Scatter plot of RP (mV) and amplitude (mV) (left) and APD 20, 50, and 90 durations (right) of WT (*n* = 18) and DMD (*n* = 7) hPSC-CCs are represented. No significant difference was observed among these parameters between WT and DMD-hPSC-CCs. Exact n value for each is represented by • in each graph. Mean ± SEM are represented. **(C)** Representative arrhythmic events recorded in WT-CCs (left) and DMD-CCs (right). Upper panels show representative recordings of non-arrhythmogenic cells from WT-CCs and DMD-CCs and bottom panels show representative recordings of arrhythmogenic cells showing extra events, such as DAD (Asterisks).

Next, we tested whether dystrophin deficiency alters the cardiac electrophysiological properties and arrhythmogenicity of single hPSC-derived CCs. Action potential (AP) properties (resting membrane potential (RP), amplitude, and duration (APD) in DMD- (26–46 days old) and WT-CCs (36–41 days old) were recorded using the patch clamp technique. Age matching of the CCss was not possible in this instance due to technical reasons as only a limited number of CCs were analyzed. Both DMD- and WT-CCs mostly exhibited triangular-shaped APs with various APD90 durations ranging from 20 to 380 ms (Figures 4A,B) similarly to WT with measured APD90 ranging from 20 to 520 ms. More occasionally, plateau-shaped APs with longer (>300 ms) APD90 durations were observed (Figures 4A,B, Supplementary Figure 7). No significant difference was observed in RP, amplitude, and durations between DMD- (*n* = 7) and WT-CCs (*n* = 18) APs (Figure 4B). Occurrence of fast pacing induced delayed afterdepolarization (DAD) events was observed and quantified. No difference (*p* = 0.25) between DMD-CCs (50%; 1.2 ± 0.3 events/10 s, *n* = 7) and WT-CCs (35%; 1.9 ± 0.4 events/10 s, *n* = 18) in the occurrence of DAD was observed (Figure 4C).

### DMD-CCs Exhibit Abnormal Intracellular Ca^2+^ Handling

Intracellular SR Ca^2+^ handling has been shown to be affected in several DMD models, in particular through RyR2 mediated Ca^2+^ leak (Andersson and Marks, 2010; Fauconnier et al., 2010; Andersson et al., 2012). Using confocal fluorescent microscopy as we previously showed in hPSC-CCs (Pesl et al., 2014; Acimovic et al., 2018), we investigated the enzymatically dissociated single cell Ca^2+^ handling in DMD and WT-CCs matched between 40 and 50 days after differentiation. We chose this age as a safe timepoint to interpret the eventual differences as prevalently caused by the DMD mutation: it is in fact reported that calcium machinery in hPSC-CCs reaches a “stationary state” between 21 and 30 days after induction (Hwang et al., 2015). We acquired intracellular Ca^2+^ transients in resting state and under physiological pacing conditions (0.5 and 1 Hz). We analyzed spontaneous frequency of calcium transients, together with their time parameters, and occurrence of secondary small diastolic calcium events possibly triggering increased automaticity.

**Figure 5:**
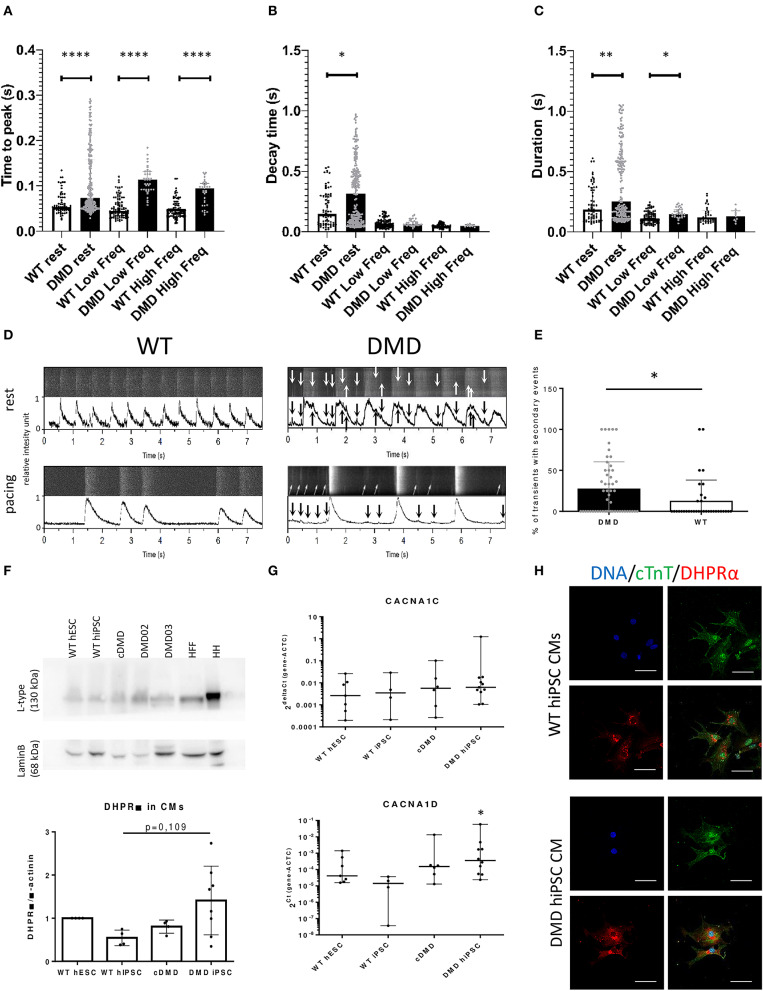
The dynamics of Ca^2+^ release and re-pumping is affected by dystrophin deficiency. Release of Ca^2+^ transients was measured using Fluo4 dyes in enzymatically dissociated DMD (91 cells and *n* = 278 transients were analyzed for resting conditions; 20 cells and *n* = 83 transients were analyzed for low frequency paced at 0.5 Hz) and WT-CCs (30 cells and *n* = 262 transients were analyzed for resting conditions; 15 cells and *n* = 128 transients were analyzed for low frequency paced at 0.5 Hz) in the age 40–50 days of differentiation. **(A)** Time to peak, **(B)** decay time, and **(C)** contraction duration time were analyzed for the main Ca^2+^ transients. The DMD-CMs showed significantly higher Ca^2+^ amplitude and longer time to release and repump/extrude the Ca^2+^ back into the SR/out of the cell in comparison to WT. The exact values are plotted in the graphs; red line represents the median. Significance (*p < 0.05) is visualized as asterisks as calculated by Mann–Whitney one-sided test (*p < 0.05, **p < 0.01, ****p < 0.0001). The Ca^2+^ leakage is demonstrated by Ca^2+^ events of low-amplitude (arrows) among the main Ca^2+^ transients [**D**—the obtained data from microscope with line scanning (upper bar) and transferred into peak graph by PeakInspector (lower bar) with and without electrical pacing of 0.5 Hz]. The occurrence of these small events is increased in DMD-CCs transients **(E)** as analyzed by Mann–Whitney test (*n* = 54 and *n* = 35 analyzed cells, for DMD and WT, respectively). **(F)** L-type channel (DHPR subunit) expression was characterized, showing non-significant increase in protein expression in DMD-hiPSC compared to WT-EBs as calculated by one-way ANOVA (*n* = 4, *n* = 4, *n* = 4 and *n* = 8 for WT hESC, WT-hiPSC, cDMD, and DMD-hiPSC-EBs). HH was used as positive control, HFF (human foreskin fibroblasts) as non-CM cell sample. **(G)** CaV isoform's (CACNA1C and CACNA1D) mRNA expression of LTCC subunits were analyzed using rt qPCR showing no difference between the groups. The values were calculated for CMs using ACTC for normalization. At least three biological repetitions were used for the analysis with exact value for each represented by • in the graph. Statistical difference was calculated using one-way ANOVA. **(H)** ICC analysis showed stronger signal for LTCC (in red) in DMD-hiPSC CCs recognized by cTnT labeling (in green). Line represents 50 μm.

A lower frequency was observed in the major Ca^2+^ transients in DMD- compared to WT-CCs in resting conditions (1.262 ± 1.608 Hz vs. 2.416 ± 3.441 Hz respectively, *p* < 0.0001). Ca^2+^ transient dynamics resulted altered in DMD single CCs: either in resting or in paced conditions, the time to peak, dependent on the SR Ca^2+^ release properties, was significantly elongated in dystrophin deficient cells (*p* < 0.0001, Figure 5A). Ca^2+^ transient decay time, expression of the features of calcium reuptake, was increased in DMD in resting state (+59.24 ms average increase, *p* = 0.013, Figure 5B); this prolongation was non-significant in pacing conditions. Transient duration, which determines the contraction cycle and possible Ca^2+^-induced arrhythmogenicity, was also elongated in DMD-CCs (Figure 5C; 63.53 ms in resting state, *p* = 0.002, and 71.51 ms under 0.5 Hz pacing, *p* = 0.025). These data suggest that the lack of dystrophin affects DMD-CCs during spontaneous and triggered SR Ca^2+^ release, affecting overall calcium dynamic.

We then observed and quantified the occurrence of secondary small-amplitude Ca^2+^ release events occurring after a main transient during diastole (relaxation and baseline, Figure 5D) and found significantly increased frequency of these events in DMD-CCs (*p* = 0.0172, Figure 5E).

### Aberrant L-Type Voltage-Dependent Ca^2+^ Channel Expression in DMD-CCs

To further understand how dystrophin deficiency affects Ca^2+^ homeostasis and ECC in DMD-CCs, we verified the expression pattern of LTCCs using dihydropyridine receptor (DHPR) α2 subunit/CACNA2D1 antibody. At the protein level, DHPR α2 subunit expression was increased in patient-specific DMD-CCs (DMD02 and 03, Figure 5F). No difference was observed on the mRNA level of the CACNA1C (encoding Cav1.2) or CACNA1D (encoding Cav1.3) between the WT and DMD groups (Figure 5G). We then tested the cellular localization of the L-type channels using DHPR immunostaining. In line with previous protein analysis, DMD-CCs (detected by cTnT labeling) showed stronger signal of the LTCC, and no other difference was detected (Figure 5H).

### DMD-EBs Display Hypocontractility, Bradycardia, and Higher Heart Rate Variability

**Figure 6:**
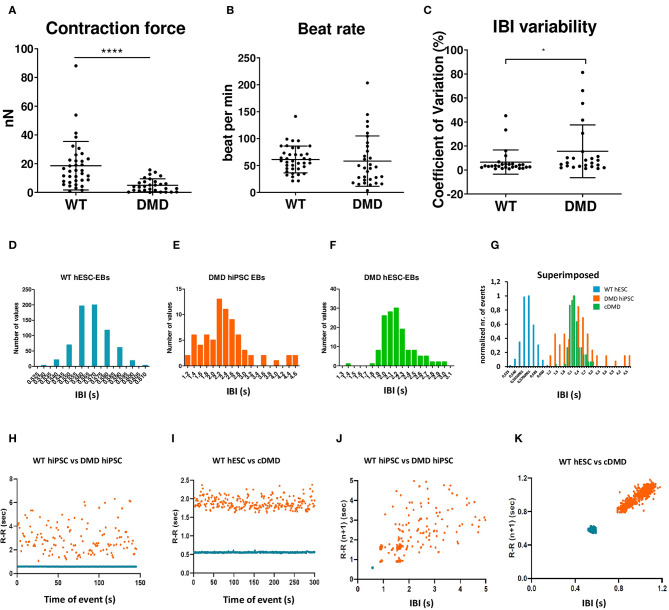
The spontaneously contracting EBs lacking dystrophin showed weaker contraction force and signs of arrhythmias. The EBs were analyzed by AFM-based methods. **(A,B)** Contraction force is decreased in DMD-hPSC derived EBs (*n* = 36 for WT, *n* = 29 for DMD-EBs), beat rate is not affected by DMD mutation (*n* = 38 for WT, *n* = 31 for DMD-EBs). The statistical difference was calculated by Mann-Whitney test (*p < 0.05, ****p < 0.0001). At least five biological repetitions were used in each differentiated line; exact value for each is represented by • in each graph. Beat rate variability was identified in DMD-EBs. Graph shows coefficient of variation analysis **(C)** of pooled control and DMD groups. The data were cleaned of outliers by ROUT test (*q* = 1%), and normality was tested by available tests showing non-normal distribution. Statistical difference was calculated using Mann–Whitney test. Representative IBI histograms of control (**D**, *n* = 712 peaks of the representative EB analyzed), DMD-hiPSC (**E**, *n* = 76 peaks of the representative EB analyzed), and DMD-hESC (**F**, *n* = 165 peaks of the representative EB analyzed) EBs and superimposed example of all three groups with normalized values **(G)**. Superimposed representative inter-beat-interval (IBI) scatter plots of control hiPSC with DMD-hiPSC-EBs (*n* = 145 time points for each EB) **(H)** and control hESC with DMD-hESC-EBs (*n* = 334 time points for each EB) **(I)**. Superimposed Poincaré plots of control hiPSC with DMD-hiPSC-EBs (**J**, same representative EBs as in **H**) and control hESC with DMD-hESC-EBs (**K**, same representative EBs as in **I**).

DMD-associated dilated cardiomyopathies is characterized by thinner left ventricle, reduced cardiac output, and hypo-contractility of the heart (Hajjar et al., 1993; Bondue et al., 2018; Amedro et al., 2019). We thus tested whether DMD-CCs contained in 3D cardiac clusters (EBs) exhibit weaker contractile properties by measuring the contraction force and beat rate by AFM as we previously published (Pesl et al., 2014, 2016; Acimovic et al., 2018). We observed weaker spontaneous contraction force in DMD-EBs (Figure 6A, *p* < 0.0001) while they exhibited similar overall beat rate compared to WT-EBs (Figure 6B, *p* = 0.139; for separate data of each line, see Supplementary Figure 6). We noticed a peculiar variability in the inter-beat interval (IBI, Figure 6C, *p* = 0.0039) (examples of AFM curves are shown in Supplementary Figure 6A) across the different samples. While WT-EBs showed consistent IBI with few exceptions, DMD-EBs showed high heterogeneity (Figure 6C, Supplementary Figures 5B–D). We observed not only different variability in recorded IBI but also prevalence in shorter IBI in WT-EBs compared to DMD-EBs (1 ± 1.0 s, and 0.96 ± 0.35 s for WT-hESC- and hiPSC-EBs respectively, 2.4 ± 1,6 s for DMD02-EBs, 2.1 ± 1.2 s for DMD03-EBs and 1.9 ± 1.2 s for cDMD-EBs, *p*-values in Supplementary Table 3) (Figures 6D–G).

Moreover, DMD-EBs displayed more dispersed IBI scatter plot and histogram than WT-EBs (Figures 6H,I). Using representative Poincaré plots, a quantitative measure of the evolvement in time of the IBI, we found more scattered inter-contraction pattern in DMD-EBs compared to WT-EBs (Figures 6J,K) showing a higher beat rate variability.

### Loss of Dystrophin Leads to β-Adrenergic Remodeling and Cardiomyocyte Wasting

**Figure 7:**
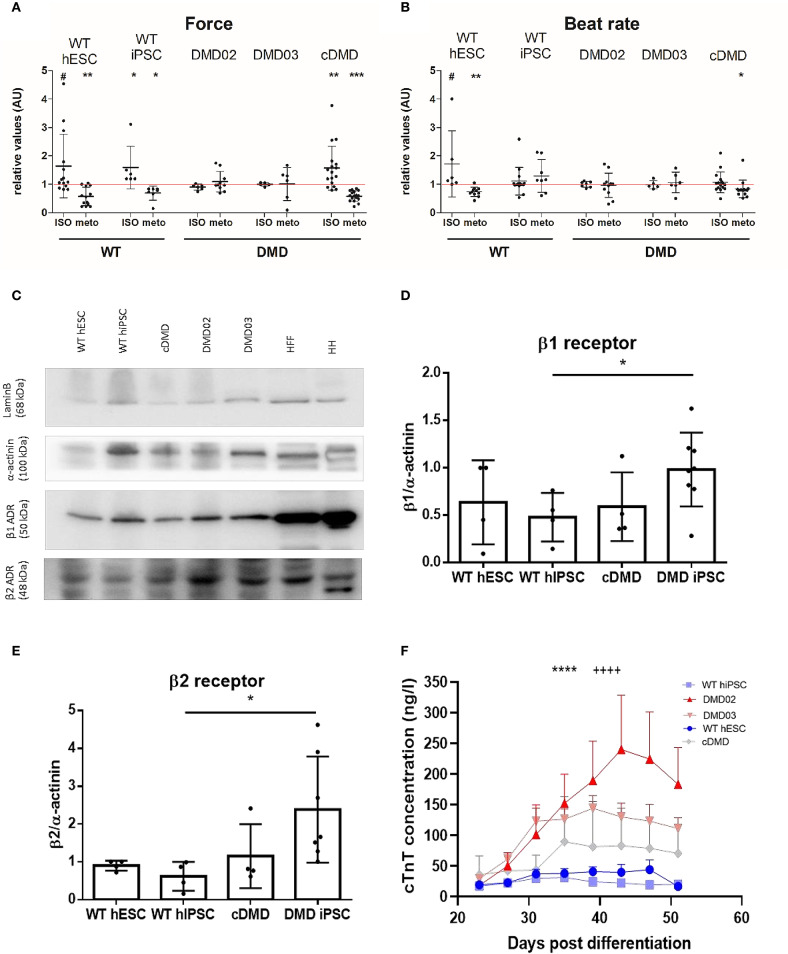
DMD-CCs show remodeled β-adrenergic response and increased death. The contraction force and beat rate were measured using an AFM-based method and tested for β adrenergic response with isoproterenol (ISO, activator) and metoprolol (METO, inhibitor) **(A)**. WT-CMs show increase in contraction force (*n* = ISO/METO: *n* = 14/13 for WT hESC, *n* = 6/7 for WT-hiPSC-EBs, *n* = 6/10 for DMD02 EBs, *n* = 5/6 for DMD03 EBs, *n* = 17/17 for cDMD-EBs, exact value for each is represented by • in each graph) and **(B)** beat rate (*n* = ISO/METO: *n* = 6/11 for WT hESC, *n* = 14/8 for WT-hiPSC-EBs, *n* = 7/11 for DMD02 EBs, *n* = 5/6 for DMD03 EBs, *n* = 15/15 for cDMD-EBs, exact value for each is represented by • in each graph) after ISO treatment and decrease in both parameters under METO treatment. The DMD-hiPSC-EBs remained unresponsive to any of these stimulations. cDMD derived EBs show similar response in contraction force to its isogenic WT control. The red line represents the ratio of 1 for better orientation in change. Significance calculated by Wilcoxon test (*p < 0.05, **p < 0.01, ***p < 0.01, ^#^*p* < 0.1) is visualized as asterisks. **(C–E)** Western blot analysis of β receptors shows higher expression of β1 in DMD-hiPSC derived EBs compared to WT-EBs; cDMD line shows similar expression to its isogenic WT control (calculates as β1 signal/actinin signal). β2 receptor showed increased levels in DMD-hiPSC-EBs. Human heart (HH) sample was used as positive control, human foreskin fibroblasts (HFF) as CM negative sample. Lamin B was used as loading control. Exact value for each is represented by • in each graph. Statistical difference was calculated by Mann-Whitney test for both β1 and β2 receptor expression. **(F)** CM death in the EBs was evaluated using free troponin level analysis in the medium. All DMD-EBs show significantly increased free cTnT levels compared to WT-EBs. Statistical difference was calculated using two-way ANOVA with uncorrected Fisher's post hoc test. ****p < 0.0001 according to cell line factor (WT vs. DMD) and ++++*p* < 0.0001 according to day of differentiation factor.

Dystrophin-deficient mouse CMs have been shown to display aberrant response to β-AR stimulation (Li et al., 2014). Thus, we evaluated the β-adrenergic response in DMD-EBs to investigate the effect of stress. We monitored the mechanobiological properties of the contracting clusters upon a selective β1-AR inhibitor (metoprolol, METO) followed by a non-selective β-AR activator (isoproterenol, ISO). In presence of METO, the WT-hPSC-EBs exhibited a significant decrease in contraction force (*p* = 0.0042 for WT hESC, *p* = 0.0156 for WT-hiPSC) and beat rate (*p* = 0.0051 for WT hESC, Figures 7A,B). DMD-hiPSC-EBs showed no response in beat rate nor contraction force to modulation by ISO nor METO. cDMD-EBs showed a response in contraction force to METO application similarly to its isogenic control (*p* = 0.0003 for contraction force and *p* = 0.103 for beat rate). WT-EBs responded to subsequent β-adrenergic stimulation by ISO application (partially competing with METO) through marginally significant increase in relative contraction force (*p* = 0.0596 for WT hESC, *p* = 0.03 for WT hiPSC, Figure 7A), DMD-EBs did not show statistically significant responses to ISO nor METO. cDMD-hESC line, however, responded similarly to its isogenic WT control in contraction force increase (with significant change in response to modulation, *p* = 0.0039). The pharmacokinetic answer to ISO has been additionally tested by exclusion of METO response using division of Tyrode and ISO value still showing response in WT-EBs (*p* = 0.0021) with no significant change in DMD-EBs (*p* = 0.1309) (as shown in Supplementary Figure 4C).

These mechanobiological data suggest that the DMD-EBs exhibit much weaker response to stress conditions in presence of β-AR modulation. Thus, we compared the expression of the β1, β2, and β3 AR at the protein level between DMD- and WT-EBs. While DMD-EBs derived from patient hiPSC showed increased β1 (*p* = 0.0103) and β2 AR expression (*p* = 0.0162) compared to isogenic WT-EBs, cDMD-EBs show similar or lower expression of both (Figures 7C–E). β3 expression was analyzed by quantitative rt qPCR showing no significant differences in between WT- and DMD-EBs (Supplementary Figure 4D).

A pathological overexpression of β-AR together with the blunted drug response and hypocontractility above described lead us to ask whether our derived CCs are already undergoing complex changes leading to their death, with consequential depletion and mechanical function deterioration in our 3D model. Thus, CC death was quantified, over time after differentiation, via cTnT concentration in the culture medium, originating from the dying CCs in the EBs. DMD lines showed from day 35 a significantly increased free cTnT level in their medium, compared to WT samples (Figure 7F, statistical evaluation in Supplementary Table 4). Interestingly, the DMD-EB kept beating even late after cardiac induction, even over the observed increased CM death (Supplementary Video 1).

## Discussion

Dilated cardiomyopathy, ultimately leading to heart failure, is the most common outcome in patients with dystrophin mutations (Kamdar and Garry, 2016; Papa et al., 2017), and it develops in DMD patients due to muscle wasting, but remains mostly unexplained in its dynamics. The DMD (*mdx)* mouse model has critical limitations since it develops only mild symptoms of heart failure with slow progression and unaffected lifespan compared to control mice (Carnwath and Shotton, 1987; Coulton et al., 1988; Shirokova and Niggli, 2013). In the present study, we provided a detailed description of patient-specific hiPSC-CCs and CRISPR/Cas9 engineered hESC-CCs. DMD-CC exhibited several molecular and functional abnormalities including an altered Ca^2+^ handling, irregular bradycardic beating, hypocontractility, and remodeling processes such as βAR alteration and CMs wasting. Our evidences on the alteration of ECC are in agreement with symptoms described in DMD patients.

Our results showed that dystrophin deficiency leads to lower efficacy in differentiating hPSCs into CCs. Interestingly, DMD-CCs exhibited a similar relative expression of known cardiac markers, and with trending increased Connexin 43 expression, similarly observed in *mdx* and *mdx:utr* CMs as a remodeling mechanism following functional dystrophin absence, with potential fatal arrhythmias in presence of β adrenergic stimulation (Patrick Gonzalez et al., 2015). Lower differentiation efficacy can point to cell fate errors: these can result from increased DNA damage caused by deregulated NOS activity (Jelinkova et al., 2019a). This process can lead to loss of stemness both at the commitment and self-renewal level (Rossi et al., 2007; Inomata et al., 2009). DNA damage can also affect multipotent stem cells division, rendering muscular differentiation ineffective (Dumont et al., 2015). Dystrophin loss was recently associated, in advanced CM *in vitro* models, with delayed contractile machinery formation, suggesting a mutation-derived impairment even at the latest stage of CM development (Pioner et al., 2020).

Then, we inquired whether and to what extent the mutation affected the single cell and EB ECC. We measured the electrophysiological properties and found similar AP features between DMD- and WT-CCs. While most of the recorded AP displayed atrial-like AP properties, our results suggested that dystrophin deficiency does not alter the electrophysiological properties of the derived hPSC-CCs. The AP durations we obtained indicate mixed population of various type of excitable cells from ventricular-like CMs (APD90 ≥ 300 ms), through atrial- and nodal-like CMs (APD90 ≥ 160 ms) and undefined/immature cardiomyocyte-like cells (APD90 < 100 ms) similarly to what others previously found (He et al., 2003; Zhu et al., 2016). Nevertheless, a significant increase of Kir2.1 mRNA expression level point to a possible alteration of APD, which results in reduced effective refractory period during diastole *in vivo*. Furthermore, regarding the voltage-gated ion channels expression, DMD-CCs exhibited increased expression of DHPRα subunit of L-type Ca^2+^ channels but no difference in Cav1.2 and Cav1.3 isoform expression. L-type Ca^2+^ channels presenting gain-of-function mechanism was reported in dystrophic hearts (Koenig et al., 2014). In a recently published work (Eisen et al., 2019), it was shown that prolongation of APD in DMD-CMs was caused by increased I_Ca, L_ density, which can be matched to prolonged diastole in DMD patients (Panovský et al., 2019). APD alterations, both at the potassium and calcium current levels, can lead to ventricular tachycardia (Choi et al., 2002; McNally et al., 2015), or premature ventricular beats, possibly degenerating into Torsades-de-Pointes ventricular fibrillations (Weiss et al., 2010).

Correlated with the mentioned calcium channels alterations, we measured the intracellular Ca^2+^ transients on dissociated CCs and observed a reduced spontaneous firing in DMD-CCs, with a significantly higher occurrence of small Ca^2+^ events in diastolic phase. For Ca^2+^ dynamics, DMD-CCs presented prolonged rising time under resting and paced conditions with high variability. The Ca^2+^ transient decay time mean was significantly longer in resting conditions for DMD-CCs, even if this difference was abolished during pacing. This led to a measured prolonged duration of the transients, except under the 1 Hz physiological pacing.

Altered Ca^2+^ transients dynamics correlate with cellular alteration of ECC, as previously shown in our work and others (Sato et al., 2007; Caluori et al., 2019; Pioner et al., 2020). In particular, longer rising time might be correlated to a state of calcium overload, derived from sarcolemmal tears (Danialou et al., 2001) and SR leakage by remodeling of RyR2 by S-nitrosylation (Fauconnier et al., 2010; Oda et al., 2015) or loss of the closed-state stabilizer calstabin2 (Fauconnier et al., 2010). RyR2 misregulation can explain also that the presence of small Ca^2+^ amplitude events by random firing is a state of overload. Prolonged cytosolic Ca^2+^, as expressed by increased duration of the transients, can contribute to arrhythmic events via inverse Na^+^-Ca^2+^-exchange activation (Voigt et al., 2012) and electromechanical dissociation. However, it should be noted that young DMD boys rarely exhibit arrhythmias (Villa Chet et al., 2015). We performed patch-clamp experiments and observed no difference in the number of arrhythmic events in WT- and DMD-CCs. In our previous work, we have shown how DMD-EBs are insensitive to positive chronotropic effect of high extracellular Ca^2+^ concentration, most likely due to low diffusion gradients in case of constant cytosolic overload (Caluori et al., 2019). Nevertheless, WT-EBs tolerated high levels of extracellular Ca^2+^ by modulating their contractile activity, while DMD-EBs presented an increased and erratic electromechanical coupling delay, together with an increased calcium reuptake time. In accordance with the presented evidences, we have previously shown how DMD-EBs are less sensitive to Verapamil L-type channels inhibition.

From the single cell electrochemical experiments, and previous results in the DMD-EB electromechanical alterations, we focused on the study of 3D mechanical behavior and its modulation. EBs showed that the overall spontaneous beat rate of WT and DMD groups did not differ at rest. However, we identified an increased heart rate variability though IBI analysis and its time-dependent correlation, similarly to Eisen et al.'s observations (Eisen et al., 2019), which also showed high heterogeneity in beat rate variability in dissociated DMD-hiPSC-CCs. Heart rate variability via L-type inactivation can be increased and even become pro-arrhythmogenic in case of spontaneous SR leakage (Johnson et al., 2013), a condition proven in the case of DMD models. The measured contraction force in DMD-EBs was significantly lowered compared to WT-EBs, as previously shown in single cell models (Pioner et al., 2020) and correlated to the ejection fraction decrease in reported patient's hearts (Corrado et al., 2002; Villa Chet et al., 2015; Panovský et al., 2019). Both lower contraction force and arrhythmic events occur in DMD patient's heart from a very young age and before dilated cardiomyopathy onset. In fact, 25% of pediatric DMD patients under 10 years old already showed decreased ejection fraction and have developed sinus tachycardia (Sadek et al., 2017). Overall, our model presents functional alterations of an early-stage cardiac manifestation of DMD.

For the first time, we further evaluated the ECC modulation abilities by pharmacological response in DMD-CCs through the β-AR response. We found an impaired response in DMD-EBs through lack of contraction force and beat rate modulation similarly to *mdx* hearts (Meyers et al., 2019). Our data showed visible changes in contraction force and beat rate in WT-EBs in the presence of isoproterenol (non-selective β adrenergic agonist) or metoprolol (β1 receptor inhibitor) but no change of both parameters in DMD-EBs. Surprisingly, the CRISPR/Cas9-induced cDMD-EBs reacted to β adrenergic modulation similarly to WT-EBs. The response might involve an unknown compensatory mechanism proper of the engineered line. As further confirmation, cDMD-CCs presented similar levels of β-AR with respect to its isogenic hESC-derived WT-CCs. In contrast, patient-specific DMD-EBs showed an increased expression in β1 and β2 receptors compared to WT-EBs. It has been previously shown that *mdx* CMs present lowered density of β1-AR (Li et al., 2014) with blunted β-adrenergic signaling pathway (Lu and Hoey, 2000; Li et al., 2014). Here, patient-specific DMD-CCs showed increased protein expression level of both, still with blunted response to adrenergic modulation.

That is also similar to non-DMD heart failure, where β1 overexpression is quite common (Engelhardt et al., 1999); trying to compensate for the weak contractility of the organ and the overactivation of the receptors then leads to receptor dysfunction in signaling, thus their insensitivity (Bristow et al., 1982; Rengo et al., 2009; de Lucia et al., 2018). The impaired contraction force observed in DMD-EBs can also be caused by β3 inhibitory function (Skeberdis, 2004). β3 normally acts as β-adrenergic break, but has been also shown to temper with mechanical properties of skeletal muscle together with nNOS expression increase leading to elevated ROS production (Puzzo et al., 2016). However, our results indicated that β3 expression was unchanged in DMD-CCs although we cannot exclude difference in β3 cellular distribution. The use of β-AR modulators has been shown to be challenging in management of cardiomyopathy in DMD patients due to severe hypotension events (McNally et al., 2015). Investigations using β blockers in DMD cardiomyopathy have turned up mixed results, with some studies demonstrating a clear benefit while others showed little effect of β-AR blockade on outcomes signaling lack of response to the modulation (Kajimoto et al., 2006; Matsumura et al., 2010; Viollet et al., 2012). The β-AR responsiveness might as well be linked to the type of mutation of the *DMD* gene, which might explain why one of our models lacked dystrophin expression yet was responsive to adrenergic modulation. Nevertheless, the beat rate and contraction force response to adrenergic modulation in patient-specific DMD-EBs suggested that the human DMD phenotype is accompanied by altered density of the β-AR on the sarcolemma and less sensitive β-AR pathway. The latter can involve a hampered lusitropic effect, resulting in a hypocontractile Ca^2+^-saturated sarcomere (Culligan et al., 2002; Asp et al., 2013).

As a further original contribution, we finally investigated whether ECC alterations and regulatory pathways remodeling were associated in the dish with CCs wasting. Serum concentration of cTnT is a routinely used marker of CMs death, especially for acute myocardial infarction, although with high sensitivity assays it is also a prognostic marker of non-ischemic cardiac diseases (Park et al., 2017). We have indeed observed how even *in vitro* early stage models of DMD undergo progressive CMs injury, with detected levels of free cTnT up to 10 times the one of age-matched control lines. Dystrophin mutations, with severe symptomatology like in Duchenne's or milder like in Becker's, are all characterized with progressive cardiac symptoms stemming from loss of CMs, remodeling of the cardiac tissue with substitutive fibrosis, and ventricular dilation leading to heart failure accompanied by non-CM cell population depletion (Pesl et al., 2020). CM death can be indirectly caused by high levels of cytosolic Ca^2+^, reportedly present in DMD through several mechanisms, which increases proteolysis and triggers mitochondrial dysfunctions with consequent ATP exhaustion (Meyers and Townsend, 2019; Tsurumi et al., 2019). Despite the fact that troponin levels measured by immunoassay methods in the media cannot define the mechanisms of cardiomyocyte injury, nor prove if the cell remains viable after such injury or became apoptotic, our findings confirmed that clinical assays can be used together with our model to follow subclinical stages of DMD cardiac involvement. Remarkably, this progressive CCs death is not correlated with loss of contractility. Pioner at al. demonstrated how up to 100 days of cultivation DMD models preserved their contractile function assuming a hypertrophic phenotype (Pioner et al., 2020). A similar mechanism could explain the similar evidence we observed in our 3D model, where CM depletion might be compensated by surviving CM hypertrophy. This evidence opens further uses of our presented model to study progressive cellular adaptation at different stages of the diseases.

## Limitations of the Study

The reported study presents several limitations. The electrophysiological analysis via patch clamp lacks a proper age matching; therefore, the highly variable parameters might have masked mutation-related changes. Furthermore, no channel-specific study was performed to make a causative link between the presented channels overexpression to the calcium handling and contractile alterations observed. We did not directly characterize the age effect on calcium handling and contractility on hPSC-CC. However, we have relied on existent information on the topic to exclude a significant contribution of incomplete maturation to the reported differences.

## Conclusions

The purpose of this work was to model some key cardiac molecular properties in DMD cardiomyopathy using DMD patient-specific and engineered CRISPR/Cas9 isogenic hPSC derived cardiac cells. Our results indicated that our DMD models in the dish are functionally affected by dystrophin deficiency and recapitulate functional defects and cardiac wasting observed in the disease. This work contributes to determine cardiac defects in DMD patients. It offers an accurate tool to study human cardiomyopathy progression and test therapies in the dish.

## Data Availability Statement

The datasets generated for this study are available on request to the corresponding author.

## Ethics Statement

The fibroblasts of two DMD patients were derived from skin/muscle biopsies with the patients informed consent and St. Anne's University Hospital (Brno, Czech Republic) Ethics Committee approval under the number 37/2011. Written informed consent to participate in this study was provided by the patient/participants legal guardian.

## Author Contributions

All the authors have contributed to the manuscript writing and corrections and were included substantially in the project preparation, conception, and evaluation. SJ has differentiated the cardiomyocytes, ran ICC, PCR, and western blot analyses, evaluated obtained data and interpreted them, prepared the figures, and wrote the draft of the manuscript. AV was included in the experimental design, differentiated cardiomyocytes and performed Ca^2+^ imaging and ICC staining, and helped interpret the data. JP performed the AFM measurements and initial evaluation of the data. FA performed the electrophysiological measurements and interpreted them. MP performed AFM measurement of the EBs. GC participated in AFM measurements and interpretation of the results. AS performed AFM and Ca^2+^ imaging data analysis. IA performed Ca^2+^ imaging of the WT cells and helped interpret the data. VS and HD performed cTnT assays. SK evaluated AFM data. TU performed dystrophin western blot. PS, AL, PD, AM, and VR supervised the project, interpreted the obtained results, and corrected the manuscript. AM and VR were responsible for the experimental design of the project, coordination, and supervision in equal matter.

## Conflict of Interest

The authors declare that the research was conducted in the absence of any commercial or financial relationships that could be construed as a potential conflict of interest.

## Abbreviations

ACTC1: cardiac muscle alpha actin
AFM: atomic force microscopy
AP: action potential
APD: action potential duration
AR: adrenergic receptor
BMP4: bone morphogenic factor 4
CC: cardiac cell
cDMD: CRISPR/Cas9 generated DMD-hESC line
cl4: control hiPSC line
CM: cardiomyocyte
cTNT: cardiac troponin T
DAD: delayed after depolarization
DAPI: 4′,6-Diamidine-2′-phenylindole dihydrochloride
DCM: dilated cardiomyopathy
DHPR: dihydropyridine receptor
DMD: Duchenne muscular dystrophy
EB: embryonic body
ECC: excitation-contraction coupling
FGF2: fibroblast growth factor (basic, or 2)
FKBP12. 6: calstabin 2
GJA1: connexin43
hESC: human embryonic stem cells
hiPSC: human induced pluripotent stem cells
hPSC: human pluripotent stem cells
IBI: inter-beat variability
ISO: isoproterenol
Kir2.1, Kir2.2, Kir2.3: potassium channels encoded by KCNJ2, KCNJ4, and KCNJ12 genes respectively
LTCC: L-type calcium channel
mdx; mdx:utr: muscular dystrophy on X chromosome, mouse model lacking dystrophin; utrophin and dystrophin lacking mouse model
mEF: mouse embryonic fibroblasts
METO: metoprolol
MYH6: myosin heavy chain α
MYH7: myosin heavy chain β
MYL2: myosin light chain 2
MYL7: myosin light chain 7
NKX2.5: NK homeobox 5
NOS: nitric oxide synthase, inducible (iNOS), neuronal (nNOS)
qRT-PCR: quantitative real-time PCR
ROS: reactive oxygen species
RyR2: ryanodine receptor 2
SAC: stretch activated channels
SERCA (ATP2A): Sarcoplasmic/endoplasmic reticulum calcium ATPase 1
SR: sarcoplasmic reticulum
VEGF: vascular endothelial growth factor
WT: wild type control.
