# Supplementary material for: DMD Pluripotent Stem Cell Derived Cardiac Cells Recapitulate in vitro Human Cardiac Pathophysiology
Source: Front Bioeng Biotechnol. 2020 Jun 19;8:535. doi: 10.3389/fbioe.2020.00535 (PMC7325914; doi:10.3389/fbioe.2020.00535)
Supplement: Supplementary file 1 [file Data_Sheet_1.docx]

Supplementary Material

# Supplementary Tables

**Table 1:** Primers

| gene | Forward primer sequence (5′–3′) | Reverse primer sequence (5′–3′) |
| --- | --- | --- |
| GAPDH | AGCCACATCGCTCAGACACC | GTACTCAGCGCCAGCATC G |
| ADRB3 | GCTCCGTGGCCTCACGAG | CTCGGCGTCGGCCCCTA |
| MYL2 | TACGTTCGGGAAATGCTGAC | TTCTCCGTGGGTGATGATG |
| ACTC1 | GCCCTGGATTTTGAGAATGA | ATGCCAGCAGATTCCATACC |
| GJA1 | AAGTACCAAACAGCAGCGGAG | ACGAAAGGCAGACTGCTCATC |
| RYR2 | CTGGTGAGGAAGAAGCCAAG | TGTCTTCCTGGCTGTGAGTG |
| MYH6 | CTCCTACGCAACTGCCGATA | GGGATGATGCAACGCAC |
| MYH7 | GAGAAACACGCAACAGAGAAC | GACCTTGTCCTCCTCGG |
| MYL 7 | CCGTCTTCCTCACGCTCTT | TGAACTCATCCTTGTTCACCAC |
| NKX2.5 | TAGAGCCGAAAAGAAAGAGCTG | TCTGGAACCAGATCTTGACCT |
| CACNA1C | GGAGAGTTTTCCAAAGAGAG | TTTGAGATCCTCTTCTAGCTG |
| CACNA1D | AAAATGGGCATCATTCTTCC | AGTTTCATAATAGCGGGTTC |
| KCNJ2 | TTGCTTTGGCTCACTCGCTT | AAACACAGCAGCCCTTACCT |
| KCNJ4 | CTCTCGTCGGACCCTCC | CTTGCCCATGCCATAAAGCG |
| KCNJ12 | CTTGGACCAGTGTCCAGCAT | CTGTCTGTCAGCACAAGCCT |
| ATP2A2 | CTTGGCTATTGGCTGTTACG | TTGCACAATCCACGCCT |

**Table 2:** Statistical difference calculated for cardiac differentiation efficacy

| Dunn's multiple comparisons test | Significant | Summary | Adjusted P Value |
| --- | --- | --- | --- |
| DMD02 vs. DMD03 | No | ns | > 0,9999 |
| DMD02 vs. CCTL14 | Yes | **** | < 0,0001 |
| DMD02 vs. CCTL12 | Yes | **** | < 0,0001 |
| DMD02 vs. CCTL13 | Yes | **** | < 0,0001 |
| DMD02 vs. cl.4 | Yes | **** | < 0,0001 |
| DMD02 vs. cDMD | No | ns | > 0,9999 |
| DMD03 vs. CCTL14 | Yes | * | 0,04 |
| DMD03 vs. CCTL12 | Yes | ** | 0,0087 |
| DMD03 vs. CCTL13 | Yes | ** | 0,0085 |
| DMD03 vs. cl.4 | Yes | ** | 0,0039 |
| DMD03 vs. cDMD | No | ns | > 0,9999 |
| CCTL14 vs. CCTL12 | No | ns | > 0,9999 |
| CCTL14 vs. CCTL13 | No | ns | > 0,9999 |
| CCTL14 vs. cl.4 | No | ns | > 0,9999 |
| CCTL14 vs. cDMD | Yes | *** | 0,0001 |
| CCTL12 vs. CCTL13 | No | ns | > 0,9999 |
| CCTL12 vs. cl.4 | No | ns | > 0,9999 |
| CCTL12 vs. cDMD | Yes | *** | 0,0003 |
| CCTL13 vs. cl.4 | No | ns | > 0,9999 |
| CCTL13 vs. cDMD | Yes | *** | 0,0002 |
| cl.4 vs. cDMD | Yes | **** | < 0,0001 |

**Table 3:** Statistical difference calculated from interbeat period of separate lines of EBs

| Games-Howell's multiple comparisons test | Significant | Summary | Adjusted P Value |
| --- | --- | --- | --- |
| iPSC vs. hESC | No | ns | 0,9873 |
| iPSC vs. DMD2 | No | ns | 0,5058 |
| iPSC vs. DMD3 | No | ns | 0,4369 |
| iPSC vs. cDMD | No | ns | 0,2237 |
| hESC vs. DMD2 | No | ns | 0,4155 |
| hESC vs. DMD3 | No | ns | 0,3201 |
| hESC vs. cDMD | No | ns | 0,0579 |
| DMD2 vs. DMD3 | No | ns | 0,9981 |
| DMD2 vs. cDMD | No | ns | 0,9678 |
| DMD3 vs. cDMD | No | ns | 0,995 |

**Table 4:** Statistical difference calculated from cTpT release from EBs

| \| Fisher´s uncorrected LSD test \| Predicted (LS) mean diff. \| 95% CI of diff. \| Significan? \| Summary \| Individual p value \| \| --- \| --- \| --- \| --- \| --- \| --- \| \| CCTL14 vs. cl4 \| 8.512 \| -16.94 to 33.97 \| No \| ns \| 0.5089 \| \| CCTL14 vs. DMD02 \| -113.1 \| -140.5 to -85.80 \| Yes \| **** \| <0.0001 \| \| CCTL14 vs. DMD03 \| -74.34 \| -99.45 to -49.23 \| Yes \| **** \| <0.0001 \| \| CCTL14 vs. cDMD \| -33.24 \| -59.18 to -7.304 \| Yes \| * \| 0.0125 \| \| cl4 vs. DMD02 \| -121.7 \| -148.1 to -95.23 \| Yes \| **** \| <0.0001 \| \| cl4 vs. DMD03 \| -82.85 \| -106.9 to -58.75 \| Yes \| **** \| <0.0001 \| |  |  |  |  |  |
| --- | --- | --- | --- | --- | --- | --- | --- | --- | --- | --- | --- | --- | --- | --- | --- | --- | --- | --- | --- | --- | --- | --- | --- | --- | --- | --- | --- | --- | --- | --- | --- | --- | --- | --- | --- | --- | --- | --- | --- | --- | --- | --- | --- | --- | --- | --- | --- |

|  |  |  |  |  |  |
| --- | --- | --- | --- | --- | --- |
|  |  |  |  |  |  |

# Supplementary Figures


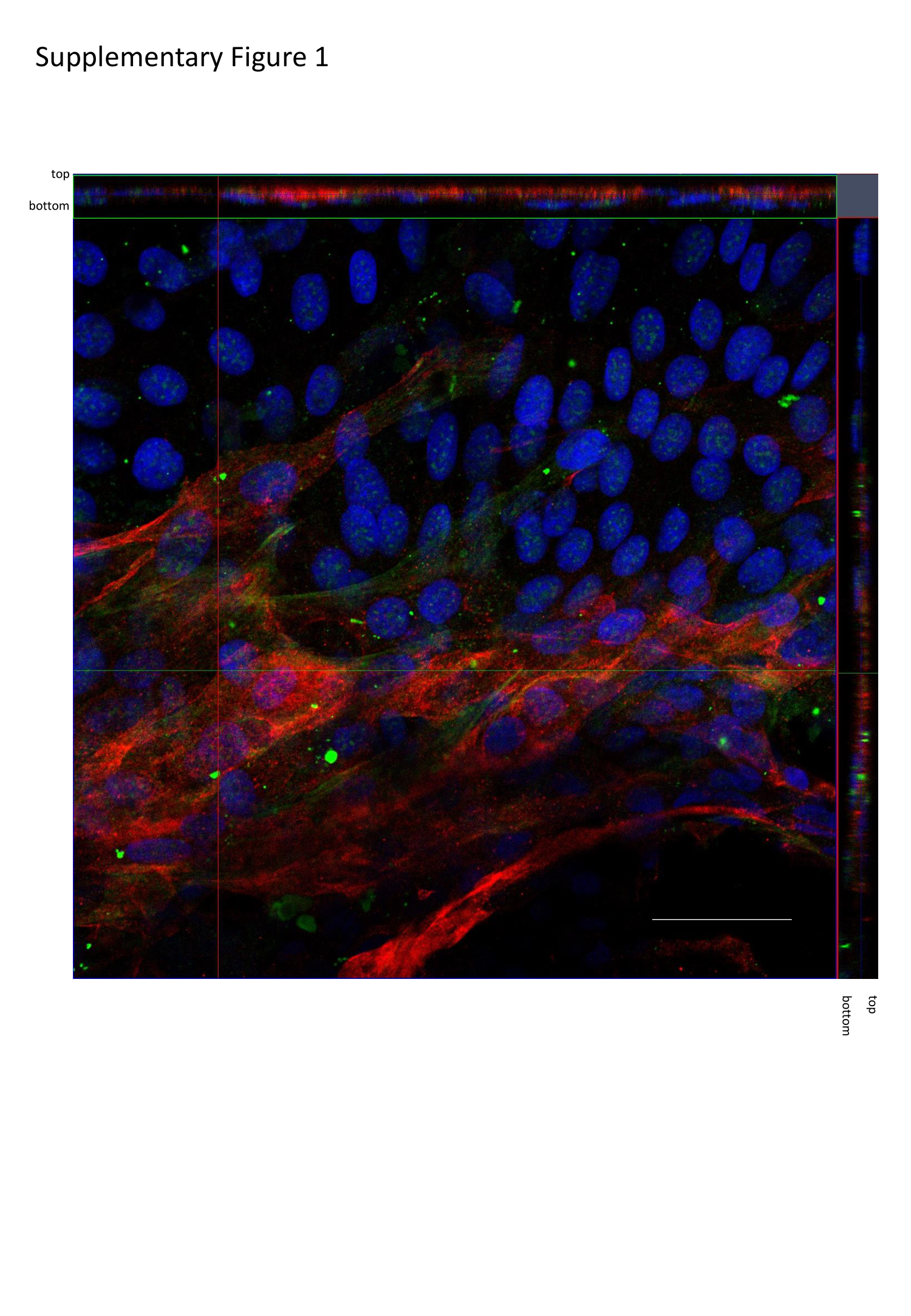
**Supplementary Figure 1:** Dystrophin is localized on the membrane of cardiomyocytes

Confocal images of dissociated EBs were taken (with distance of 0,2 um for layer) and orthographic image of protein localization was made. Nuclei (blue) are localized on the bottom of the dish, cardiac troponin T (green) in the cytoplasm, dystrophin (red) is localized above the nuclei. Line represents 50 μm.



**Supplementary Figure 2:** (A) Full membrane of N-terminus detecting dystrophin antibody with uncorrected exposure for the samples and ladder photographed in the visible light overlaying the membrane. (B) Full membrane of C-terminus detecting dystrophin antibody with uncorrected exposure for the samples and ladder photographed in the visible light overlaying the membrane.



**Supplementary Figure 3:** Full membrane (in bright field upper panel, exposed membrane lower panel of LTCC detecting antibodies with laminB as loading control and actinin as marker of CMs.

**
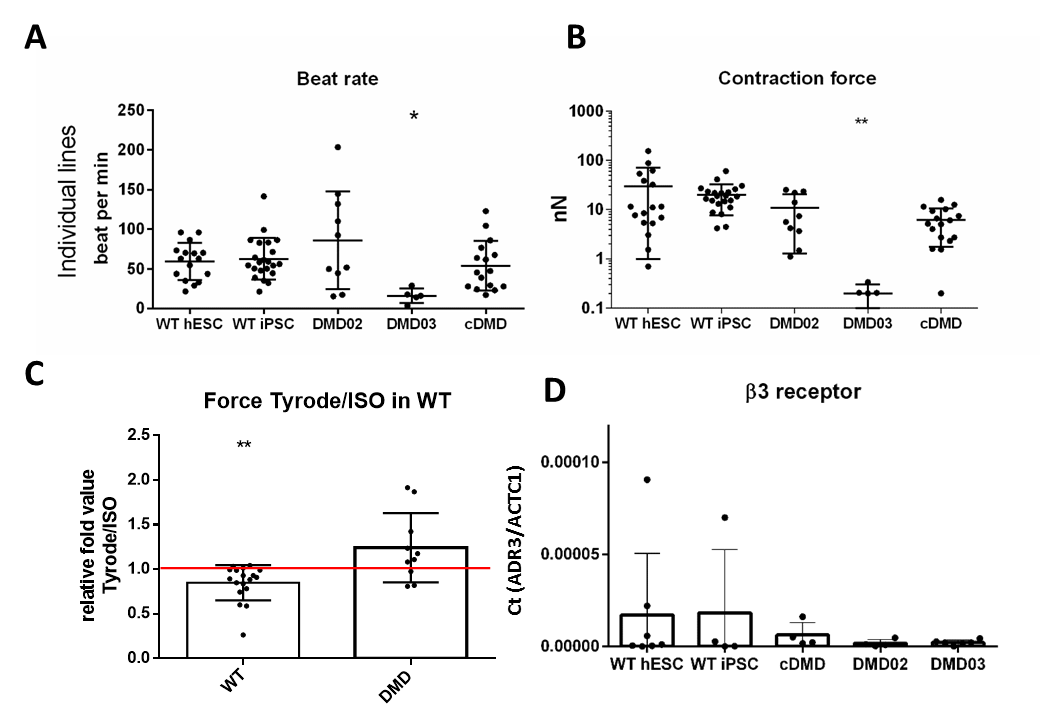
Supplementary Figure 4:** Individual lines analysis of contraction force and beat rate. The EBs analyzed by AFM-based methods show lower contraction force (A) in DMD hPSC derived EBs, beat rate (B) is not affected by DMD mutation (only line specific) (n=17 WT hESC EBs, n=22 WT hiPSC EBs, n=10 DMD02 EBs, n=5 DMD03 EBs and n= 17 cDMD EBs). The statistical difference was calculated by two-way ANOVA and compared to WT hPSC CMs (*p<0.05, **p<0.01). Exact value for each EB is represented by • in each graph. Beat rate variability was identified in DMD-EBs. (C) The contraction force was measured using an AFM-based method and tested for β adrenergic response with isoproterenol (ISO, activator) applying division of Tyrode/ISO to exclude the effect of METO on the EBs. The DMD hiPSC EBs remained unresponsive to any of these stimulations. While WT EBs show significant increase in contraction force with ISO treatment. The red line represents the ratio of 1 for better orientation in change. Significance calculated by Wilcoxon test (**p<0.01) is visualized as asterisks. (D) β3 receptor expression was analyzed using quantitative rtPCR showing no significant difference in expression levels of the β3. Statistical difference was calculated by Kruskall-Wallis test correction and Dunn´s post hoc test (n=7 for WT hESC, n=4 for WT hiPSC, n=4 for cDMD, n=6 for DMD02 amnd n=6 for DMD03 EBs).


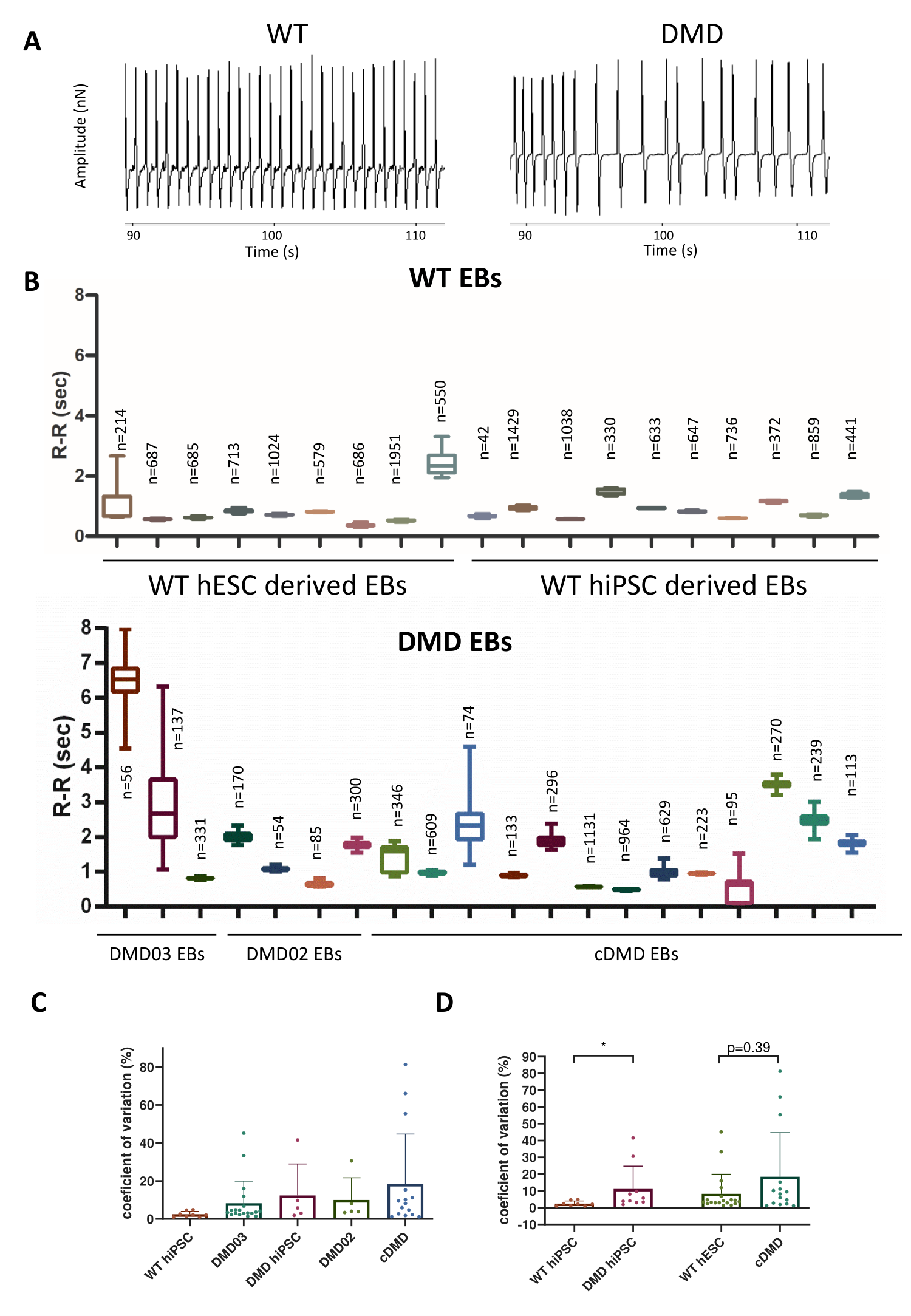
**Supplementary Figure 5:** (A) Examples of AFM recordings from WT and DMD-EB showing homogenous pattern of beating in the WT-EB and different length of time between peaks in DMD. Analyzed WT-EBs (B upper panel) on AFM show homogeneous IBI with low variability of data while DMD-EBs (B lower panel) show high variability in recordings of IBI (calculated as R-R distance from the AFM curve, box represents 25-75% of data recorded, whiskers show lower and higher 25% of the measured data, each column represents individual EB, n values for each column is noted in the graph). (C) Analysis of standard deviations in separate cell line groups of EBs show lower variability in WT lines and high variability in all DMD lines differentiated into contracting EBs (n=19 fo hESC, n=8 for hiPSC, n=5 for DMD02, n=5 for DMD03 and n=15 for cDMD EBs). Statistical difference was calculated by two-way ANOVA. (D) Groups were analyzed based on their background and WT and cDMD lines were compared together, WT and DMD hiPSC were compared together (Mann Whitney test was used for calculation of statistical difference. *p<0,05, n=8 for WT hiPSC, n=10 for DMD hiPSC, n=28 for WT hESC and n=15 for cDMD EBs).



**Supplementary Figure 6:** Full membrane (in bright field upper panel, exposed membrane lower panel of βAR detecting antibodies with laminB as loading control and actinin as marker of CMs.


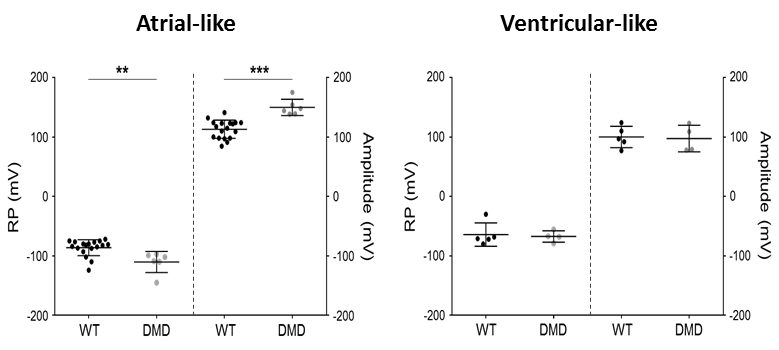


**Supplementary Figure 7:** We recorded AP with various duration in our conditions. Among these cells, most of them are triangular shaped with short duration (shown as most representative in our study in Fig. 4). Some of them exhibit longer plateau shape. Since ventricular AP in human are close to 400 ms, we arbitrary decided to separate two population of cells by using APD90>300 ms.
